# Evaluation of a Novel Ear Pulse Oximeter: Towards Automated Oxygen Titration in Eyeglass Frames

**DOI:** 10.3390/s20113301

**Published:** 2020-06-10

**Authors:** Fabian Braun, Christophe Verjus, Josep Solà, Marcus Marienfeld, Manuela Funke-Chambour, Jens Krauss, Thomas Geiser, Sabina A. Guler

**Affiliations:** 1 Centre Suisse d′Electronique et de Microtechnique (CSEM), CH-2000 Neuchâtel, Switzerland; cve@csem.ch (C.V.); josep.sola@aktiia.com (J.S.); jkr@csem.ch (J.K.); 2 Marcus Marienfeld AG, CH-3911 Ried-Brig, Switzerland; info@marienfeld.ch; 3 Department of Pulmonary Medicine, Inselspital, Bern University Hospital, University of Bern, CH-3010 Bern, Switzerland; manuela.funke-chambour@insel.ch (M.F.-C.); Thomas.Geiser@insel.ch (T.G.)

**Keywords:** pulse oximetry, cavum conchae, long-term oxygen therapy, hypoxemia, interstitial lung disease, chronic obstructive pulmonary disease, quality of life

## Abstract

Current oxygen delivery modes lack monitoring and can be cumbersome for patients with chronic respiratory diseases. Integrating a pulse oximeter and nasal oxygen cannulas into eyeglasses would reduce the burden of current solutions. An ear pulse oximeter (OxyFrame) was evaluated on 16 healthy volunteers and 20 hypoxemic patients with chronic respiratory diseases undergoing a prespecified protocol simulating daily activities. Correlation, error, and accuracy root mean square error (A_RMS_) were calculated to compare S_p_O_2_ measured by OxyFrame, a standard pulse oximeter (MASIMO), and arterial blood gas analysis (aBGA). S_p_O_2_ measured by OxyFrame and MASIMO correlated strongly in volunteers, with low error and high accuracy (r = 0.85, error = 0.2 ± 2.9%, A_RMS_ = 2.88%). Performances were similar in patients (r = 0.87, error 0 ± 2.5%, A_RMS_ = 2.49% compared with MASIMO; and r = 0.93, error = 0.4 ± 1.9%, A_RMS_ = 1.94% compared with aBGA). However, the percentage of rejected measurements was high (volunteers 77.2%, patients 46.9%). The OxyFrame cavum conchae pulse oximeter was successfully evaluated, and demonstrated accurate S_p_O_2_ measurements, compliant with ISO 80601-2-61:2017. Several reasons for the high rejection rate were identified, and potential solutions were proposed, which might be valuable for optimization of the sensor hardware.

## 1. Introduction

Long-term oxygen therapy (LTOT) is commonly used for patients with chronic obstructive pulmonary disease (COPD) and interstitial lung disease (ILD), who suffer from the consequences of chronic hypoxemia. LTOT improves quality of life, physical performance, dyspnea, and survival, and is recommended for the treatment of severe resting hypoxemia in patients with COPD [1,2]. The role of LTOT during physical activity in patients with ILD is an active field of research [3,4,5].

The accurate measurement of blood oxygenation is needed to monitor hypoxemia in patients who are candidates for LTOT, or who are already being treated with LTOT. Pulse oximetry is widely used for non-invasive assessment of blood oxygenation in-hospital, for outpatient assessment, for the diagnosis of sleep related breathing disorders, and recently for wearable health monitoring technology [6]. Pulse oximeters are most frequently applied on fingers or earlobes, where a light source (LED) is placed on one, and a light detector on the other side of the probed tissue [7]. Other potential application sites include the forehead, pharynx, esophagus, trachea, the nasal cavity, and the ear canal [8,9,10,11,12,13,14,15,16]. Electronic amplification and recent progress in digital processing and management of bigger data volumes allow the acquisition of cleaner, more reliable signals, at higher sampling rates. However, most pulse oximeters are not validated to measure severe hypoxemia, and frequent motion artefacts make the estimation of blood oxygenation at ambulation challenging. Furthermore, ambient light can interfere with the measurements, and pathophysiological variabilities, such as fluctuating perfusion, body temperature, and cardiac output, demand the validation of pulse oximeters outside laboratory conditions [17].

**Figure 1:**
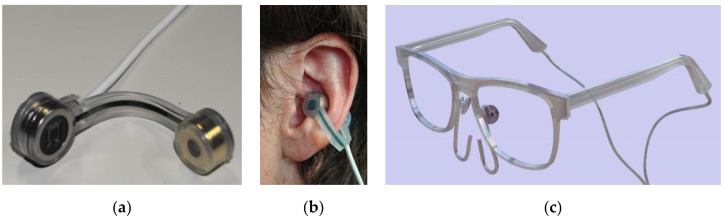
The sensor earpiece of the OxyFrame device in: (**a**) detached state showing the photodetector and the LEDs, and (**b**) attached at the cavum conchae of a volunteer’s ear holding together with statics magnets embedded in the sensor. (**c**) Prototype oxygen titration eyeglasses with integrated nasal oxygen cannulas (only for illustration of the future application).

We aimed to develop a pulse oximeter for future integration into the frames of daily use eyeglasses, which also contain nasal cannulas for oxygen delivery (Figure 1c). Such a new pulse oximeter needs to reliably measure the fluctuating blood oxygenation during common activities of daily living in LTOT dependent patients.

Herein we report on the design, evaluation, and future applications of the OxyFrame cavum conchae pulse oximeter.

## 2. Materials and Methods

### 2.1. Study Participants and Protocol

The study protocol (ClinicalTrials.gov NCT02723032) has been approved by Swissmedic (no. 2015-MD-0028, CIV-15-10-013926) and by the local ethical committee (Swiss Ethics Committee, Bern, Switzerland, no. 312/15).

#### 2.1.1. Healthy Volunteers

For the first phase of the study, healthy volunteers were invited to participate. Non-smokers of good general health and physical fitness were chosen as volunteers; subjects with significant heart, lung, neurological, or metabolic diseases and anemia were excluded from participation.

The study protocol included a normobaric hypoxemic challenge test (AltiTrainer^®^, SMTEC, Nyon, Switzerland) with participants wearing a facemask and breathing a gas mixture containing a fraction of inspired oxygen (FiO_2_), which was reduced in a stepwise manner. The initial FiO_2_ of 21% was reduced to a minimum of 10.5%. Participants simulated usual daily activity by walking on place, sitting, standing, and lying for 3 subsequent minutes at each level of oxygenation, including normoxemia, mild hypoxemia (peripheral capillary hemoglobin oxygen saturation [S_p_O_2_] 85–92%) and severe hypoxemia (S_p_O_2_ 80–85%). Oxygen saturation was measured constantly by a standard pulse oximeter (MightySat Rx^®^, Masimo, Irvine, CA, USA) and by the OxyFrame device.

#### 2.1.2. Patients

Included subjects with COPD and ILD are diagnosed and treated according to current guidelines [18,19,20]. Patients qualifying for LTOT at rest (p_a_O_2_ ≤ 55 mmHg or <60 mmHg with pulmonary hypertension, congestive cardiac failure, or polycythemia) and/or on physical activity (exercise S_p_O_2_ < 90%) were recruited from our in- and outpatient clinic for pulmonary medicine. Main exclusion criteria were severe hypoxemia or hypercapnia (p_a_O_2_ < 40 mmHg and/or p_a_CO_2_ > 55 mmHg), severe neurologic, metabolic or heart disease, anemia (hemoglobin < 120 g/L), and current smoking.

A sample size of 14 subjects was calculated to achieve a statistical power of 90% and α of 0.05 (confidence interval of 95%), considering a 2% difference of S_p_O_2_ being clinically significant, and a standard deviation of maximal 1.8 to be expected [13]. Accounting for possible dropout, the inclusion of 20 patients in the study was intended.

Patients performed a uniform, pre-specified protocol breathing room air or additional nasal oxygen (0–6 L/min), depending on their actual needs and individual symptoms. Patients were instructed to perform a sequence of specific positions reflecting their daily activities: lying, sitting, standing, walking (6-min walking test, 6MWT), and cycling. Arterial blood gas analysis (aBGA) was performed at rest and during cycling (ergometer at constant workload of 15 watt). The patients’ S_p_O_2_ and heart rate were registered constantly and simultaneously by OxyFrame and a standard pulse oximeter (MightySat Rx^®^, Masimo, Irvine, CA, USA).

### 2.2. Sensor Electronics and Data Processing

The OxyFrame device shown in Figure 1a,b was designed and manufactured to record physiological signals at the cavum conchae in transmission mode. The cavum conchae is an optimal pulse oximetry body site for several reasons: The proximity of the ear to the trunk makes it less susceptible to centralization and poor peripheral perfusion artifacts, detection of desaturations might be faster, and its location being closer to the brain probably enables a more accurate reflection of cerebral oxygenation [15]. Further, at ambulation, movement of the head is less pronounced than movement of the arms so that motion artefacts can be reduced.

**Figure 2:**
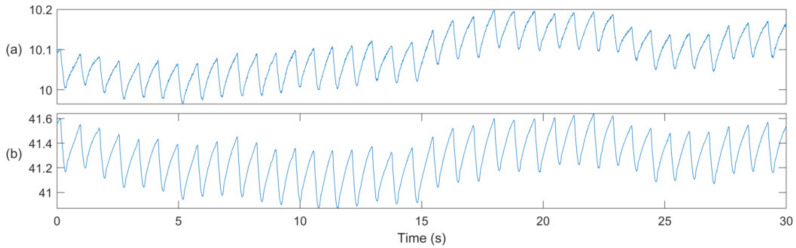
Examples of photoplethysmographic (PPG) signals at (**a**) the red (PPG-R) and (**b**) the infrared wavelength (PPG-IR). Note that the amplitude of these signals is in arbitrary units.

The device was placed at the left ear where two photoplethysmographic (PPG) signals were recorded at the infrared (PPG-IR at 940 nm) and at the red wavelength (PPG-R at 660 nm). An example of these signals is shown in Figure 2. Ambient light signals were recorded to correct PPG-R and PPG-IR signals by suppressing perturbations, due to ambient light fluctuations. The entire set of signals was further analyzed offline using MATLAB (Mathworks, Natick, MA, USA), in order to derive S_p_O_2_ estimates, according to the algorithm reported by Proença et al. [21], and briefly described in the following paragraph.

First, the alternating (AC) signal component was estimated by filtering the PPG signals with a fourth-order bandpass filter. Then, both AC and average (DC) signal components were averaged over time windows of 40 s, using cardiac-gated averaging. The ratio of signals (ROS) was estimated as ROS = (AC_PPG-R_/DC_PPG-R_)/(AC_PPG-IR_/DC_PPG-IR_), and was further transformed into S_p_O_2_ values, as described later. In addition, this algorithm provides a signal quality index (SQI), which is useful to reject unreliable measurements. An SQI lower than 70% was considered unreliable, and the corresponding measurements were excluded from analysis.

### 2.3. OxyFrame S_p_O_2_ Performance Evaluation

The performance of OxyFrame-derived S_p_O_2_ values was evaluated as recommended by the ISO 80601-2-61:2017 standard for pulse oximeters and sensors [22], time windows without stable standard S_p_O_2_ measurements were discarded prior to analysis. For the remaining measurement points the ROS of each subject were transformed into S_p_O_2_ values (expressed in %) via a linear calibration function: S_p_O_2_ = a ROS + b. The two calibration coefficients (a and b) were determined for each subject individually, by using a leave one out calibration, i.e., by using the measurements of all other subjects for calibration, while excluding the ones of the current subject. This procedure was performed independently for the two populations (volunteers and patients). This led to the following distribution of correlation coefficients: a = −29.6 ± 0.8 and b = 119.9 ± 0.8 and a = −26.8 ± 0.3 and b = 116.9 ± 0.3, in volunteers and patients, respectively.

Mean, standard deviation, Pearson’s correlation coefficient (r), error, precision, and accuracy root mean square error (A_RMS_) were calculated, and the Bland-Altman analysis was used to compare S_p_O_2_ measured by the novel sensor with the standard sensor and arterial blood samples.

## 3. Results

### 3.1. Healthy Volunteers

**Figure 3:**
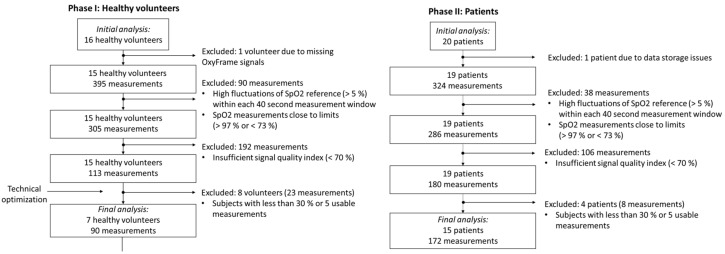
Flow chart of the two-phased study showing the number of measurements remaining for analysis, after applying specific exclusion criteria for both healthy volunteers (in phase I) and patients (in phase II).

**Figure 4:**
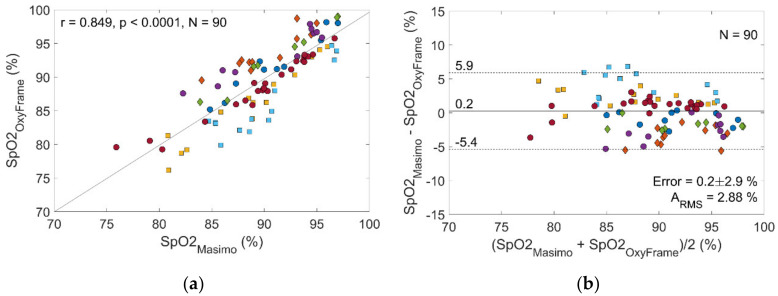
Performance of the OxyFrame device compared to a standard sensor (MASIMO) in healthy volunteers. (**a**) Correlation plot S_p_O_2_ measurement by OxyFrame versus MASIMO. (**b**) Bland-Altman plot S_p_O_2_ measurement by OxyFrame versus MASIMO. Measurements of the same volunteer have the same color and shape.

Eight men and eight women volunteered to participate in phase I of the study. The median age of the volunteers was 34.2 (range 21–57 years). The mean (SD) S_p_O_2_ and heart rate at rest were 97.4 % (1.1) and 69.8 (6.9) bpm, the mean (SD) systolic and diastolic blood pressure were 130 (16.9) mmHg and 76 (12.6) mmHg, respectively. After completing the study procedure, nine volunteers were excluded from the final analysis. For one volunteer, the optical signal was missing, due to a technical problem with the sensor data acquisition. The other eight volunteers provided a large proportion of measurements that were rejected, due to a low SQI. The major reason for this high rejection rate was a technical defect of the optical sensor earpiece, which was detected at the interim analysis of phase I and resolved before phase II of the study (evaluation in patients). Finally, 90 data points from seven volunteers were retained and analysed (Figure 3). This results in an overall rejection rate of 77.2%. Figure 4 compares S_p_O_2_ measurements obtained by OxyFrame with S_p_O_2_ measurements from the reference device (MASIMO). Data points from both devices correlated strongly (r = 0.85, S_p_O_2_ error 0.2% (standard deviation [SD] 2.9%), with an accuracy of A_RMS_ = 2.88%. When performing a separate analysis for walking and stationary sequences accuracies of A_RMS_ = 2.99% and A_RMS_ = 2.87% were obtained (Table 1). All A_RMS_ were below 4% as required by the ISO standard 80601-2-61:2017.

### 3.2. Patients

Nine patients with COPD and eleven patients with ILD consented to participate in the study. Patients’ baseline characteristics including disease severity and LTOT requirements are summarized in Table 2.

One patient was excluded from analysis due to corrupted data of the OxyFrame device, which led to the loss of data from the entire recording. Another four patients were excluded, because too few measurements of sufficient signal quality were recorded (Figure 3). Of the remaining 15 patients, 172 measurements were available for final analysis. This results in an overall rejection rate of 46.9%.

**Figure 5:**
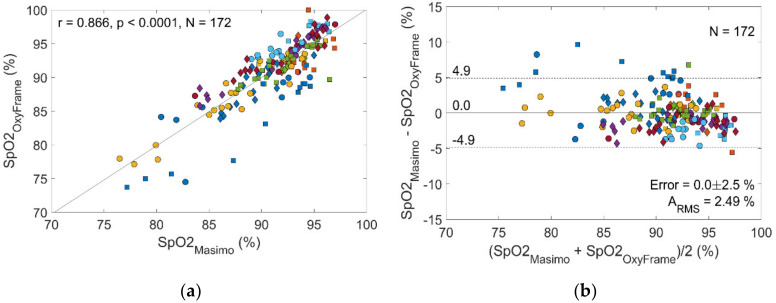
Performance of the OxyFrame device compared to a standard sensor (MASIMO) in patients. (**a**) Correlation plot S_p_O_2_ measurement by OxyFrame versus MASIMO. (**b**) Bland-Altman plot S_p_O_2_ measurement by OxyFrame versus MASIMO. Measurements of the same patient have the same color and shape.

Correlation of OxyFrame S_p_O_2_ measurement with corresponding reference measurements (MASIMO) was strong (r = 0.87, Figure 5), with low S_p_O_2_ error (0% [SD 2.5%]) and high accuracy (A_RMS_ 2.49%). When performing a separate analysis for walking and non-walking sequences accuracies were A_RMS_ 3.65% and 2.17%, respectively (Table 1).

**Figure 6:**
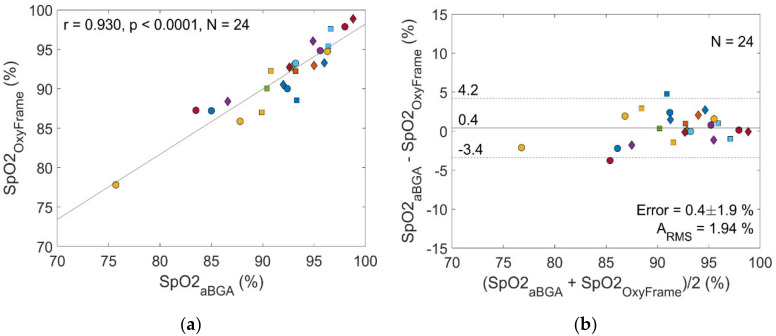
Performance of the OxyFrame device compared to arterial blood gas analysis in patients. (**a**) Correlation plot S_p_O_2_ measurement by OxyFrame versus arterial blood gas analysis (aBGA); (**b**) Bland-Altman plot S_p_O_2_ measurement by OxyFrame versus aBGA. Measurements of the same patient have the same color and shape.

S_a_O_2_ measurements from aBGA were compared to OxyFrame- and MASIMO-derived S_p_O_2_. Among the 36 initially available aBGA measurements, only 24 could be used for comparison, since 12 of the corresponding OxyFrame measurements showed insufficient signal quality. Comparing these remaining OxyFrame S_p_O_2_ measurements to the corresponding S_a_O_2_ from aBGA, the following performance characteristics were be obtained (Figure 6): r = 0.93, S_p_O_2_ error 0.4% (SD 1.9%) and A_RMS_ 1.94%. In contrast, comparing MASIMO derived S_p_O_2_ to arterial S_a_O_2_ measurements resulted in a weaker correlation, higher error, and lower accuracy (r = 0.83, S_p_O_2_ error 0.7% [SD 2.9%], A_RMS_ 2.95%).

All analyses showed an overall accuracy of A_RMS_ < 4%, as required by the ISO standard 80601-2-61.

## 4. Discussion

We demonstrate high accuracy of an ear pulse oximeter (OxyFrame) in healthy volunteers, and in patients with severe chronic respiratory diseases. This novel sensor was evaluated in a two-phase approach, according to the most recent ISO standard: after evaluation in healthy subjects undergoing a normobaric hypoxemic challenge test, we evaluated OxyFrame in LTOT dependent patients, simulating common activities of daily living. Comparison of OxyFrame with the reference pulse oximeter, and with arterial oxygen saturation, resulted in a strong correlation between corresponding measurements, with low error and high accuracy. With A_RMS_ < 4% for all analyses OxyFrame qualifies as an accurate pulse oximeter for use in healthy individuals, as well as in severely impaired patients.

### 4.1. Low Signal Quality and High Rejection Rates

A high number of measurements were automatically rejected due to insufficient signal quality (low SQI), leading to a rejection rate of 77.2% in healthy volunteers and 46.9% in patients (Figure 3). This high rejection rate successfully validates the sensitivity of our SQI algorithm, which automatically detects signals with low quality, due to the absence of physiological pulsations in the PPG signals.

There are several potential issues likely responsible for the signal quality of the OxyFrame sensor. In the interim analysis of phase I of the study, we identified a technical defect of the optical sensor earpiece, which led to the exclusion of measurements from eight volunteers before the sensor was optimized for evaluation on patients in phase II of the study. In patients, the rejection rate is lower but still significant (Table 1). This rejection is hypothesized to be mainly due to motion artefacts, which potentially deteriorate the signal quality. To explore this issue, sensitivity analyses for walking and non-walking sequences were performed, which confirmed a more challenging S_p_O_2_ measurement when subjects were walking, as indicated by a lower A_RMS_ and a higher rejection rate (Table 1). The low signal quality and the resulting high rejection rate is a problem that needs to be addressed further. However, according to ISO 80601-2-61:2017 OxyFrame measurements were still sufficiently accurate when patients were walking (A_RMS_ < 4%).

### 4.2. Issues and Potential Solutions Related to the OxyFrame Sensor

The following limitations need to be improved in a future version of the OxyFrame sensor.

First, the signal quality varies depending on the placement of the sensor earpiece. Moving the sensor to a slightly different location has shown to sometimes improve the signal quality. Thus, we suggest probing a larger area of tissue by using a photodetector with a larger surface and/or multiple LEDs for each wavelength (red and infrared). Second, the cables of the sensor earpiece can pull the sensor leading to motion artefacts during movement. We attached these cables behind the neck of the subjects, which might not have been sufficient to prevent disruption of the PPG signal. Potential solutions include the direct attachment of the sensor to eyeglass frames via a short cable, or the use of wireless data and power transmission, via a second system attached to the eyeglass frames. Third, the PPG acquisition hardware used in the OxyFrame earpiece sensor is built using discrete components. In contrast, the use of a more recent acquisition hardware with an integrated analogue front-end has shown promise to measure S_p_O_2_ [23]—even in more challenging reflection mode, PPG—and should therefore be tested in a future design.

These suggestions should be considered for a future version of the OxyFrame sensor hardware to increase its reliability in terms of signal quality when used in the daily life of LTOT dependent patients. Future validation should include a large group of patients with different respiratory diseases and oxygen requirements in diverse ambulatory settings, with testing for longer periods. This would allow confirming usability and acceptability of OxyFrame in real-life settings.

### 4.3. Clinical Perspectives

Considering its frequent use, there is surprisingly little evidence supporting the benefit of LTOT in patients with COPD and ILD [24,25,26,27,28]. Specifically, the uncertain effectiveness of LTOT in exercise induced hypoxemia, might partly be due to an insufficiently controlled and individualized mode of oxygen delivery. Despite LTOT, some patients show transient oxygen desaturation while walking [29], which likely reflects inaccurate oxygen titration in some cases. In addition to preventing hypoxemia at physical activity, it is also important not to overdose oxygen, which can cause hypercapnia, typically in COPD patients with hypoxic regulation of ventilation [30,31]. Other potentially detrimental effects of hyperoxia, such as increased oxidative stress, are not yet fully understood [32].

The OxyFrame pulse oximeter was designed for oxygen titration in patients with chronic respiratory diseases, and specifically for the future integration into eyeglass frames, including an oxygen delivery system. Automated oxygen titration systems aim to provide patients with the oxygen flow that matches their instantaneous demand [33,34,35,36]. These systems might prevent hyper- and hypoxemia and, at the same time, save oxygen resources. Lower oxygen consumption results in a larger range of motion for the patient, and lower LTOT related healthcare costs. However, safe automated oxygen titration requires pulse oximeters with high performance and accuracy, with most currently available pulse oximeters likely not meeting these criteria [37,38]. In the current study, we demonstrate the accuracy of our device. However, the reliability in terms of signal quality is not yet fully satisfactory, and a redesign of the sensor hardware is required to improve the signal quality, particularly during movement.

Besides the technical issues, patients experience a variety of practical and psychosocial challenges when using LTOT, and frequently the overall benefit from the treatment does not meet their expectations [39]. Some patients decline to use their oxygen in public, because they feel stigmatized by the cumbersome nasal cannulas, which can burden their social life, interaction, and mobility [39]. The OxyFrame pulse oximeter was designed for the future integration into eyeglass frames that dissimulate the nasal oxygen cannula, which markedly improves the wearability of the small device and the entire oxygen delivery system.

## 5. Conclusions

The OxyFrame ear pulse oximeter placed on the cavum conchae was evaluated successfully according to the stringent ISO guidelines. We demonstrated a high accuracy in healthy volunteers, and a population of patients with advanced chronic respiratory diseases simulating daily activities. However, the large number of automatically rejected measurements highlights the potential of optimization of the OxyFrame sensor hardware to increase its reliability in terms of signal quality. We strive towards an integration of the sensor into an automated closed-loop oxygen delivery system for a safer, more cost-effective, and socially acceptable mode of oxygen delivery for LTOT dependent patients.

## Figures and Tables

**Table 1:** Performance of OxyFrame compared to a standard pulse oximeter (MASIMO) for walking and stationary activities. The error values are shown as mean (standard deviation).

	Volunteers			Patients		
	Overall	Walking	Stationary	Overall	Walking	Stationary
Error (%)	0.2 (2.9)	1.5 (2.8)	0.1 (2.9)	0.0 (2.5)	1.6 (3.4)	0.3 (2.2)
r	0.849 *	0.901 ^†^	0.845 *	0.866 *	0.829 *	0.804 *
A_RMS_ (%)	2.88	2.99	2.87	2.49	3.65	2.17
Rejection Rate (%)	77.2	92.4	72.6	46.9	71.3	35.9

* *p* < 0.001, ^†^ *p* < 0.01; r: correlation coefficient; A_RMS_: accuracy root mean square error.

**Table 2:** Baseline characteristics of participating subjects in the patient group (phase II of study). Values shown represent mean (standard deviation) or median (range)**.**

	All (n = 20)	COPD (n = 9)	ILD (n = 11)
	Demographics		
Age, years	65.9 (55–72)	66.6 (59–72)	65.4 (55–72)
Male/female	15/5	7/2	8/3
BMI, kg/m^2^	26.1 (5.9)	23.8 (7.0)	28.1 (3.8)
Smoked pack-years	46 (36)	75 (31.6)	20 (11.9)
	Pulmonary Function Test		
TLC, % predicted	85.5 (31.6)	123.3 (15.4)	64.8 (14.2)
FEV1/FVC, %	62.2 (21.4)	40.9 (10.9)	79.6 (7.7)
FVC, % predicted	64.1 (17.6)	66.1 (17.6)	62.5 (17.4)
FEV1, % predicted	51.5 (24.3)	34.9 (15.3)	65.0 (21.8)
DLCO, % predicted	41.0 (14.7)	37.6 (9.4)	43.8 (17.3)
	6-min Walking Test		
6MWD, meters	366 (119)	327 (126)	387 (108)
6MWD, % predicted	69 (20.7)	65.2 (23.6)	73.9 (16.6)
S_p_O_2_ at rest	91.8 (3.7)	92.8 (3.7)	90.9 (3.5)
S_p_O_2_ nadir	84 (5.6)	87.1 (3.5)	82 (6.1)
O2 for 6MWT, yes/no	12/8	6/3	6/5
O2, l/min	3.7 (1.5–6)	3.6 (1.5–6)	3.8 (2–6)

BMI: body mass index; DLCO: diffusing capacity of the lung for carbon monoxide; FEV1: forced expiratory volume in 1 s; FVC: forced vital capacity; LTOT: long term oxygen therapy; TLC: total lung capacity; 6MWD: 6-min walk distance.
